# Functional adaptation of cortical interneurons to attenuated activity is subtype-specific

**DOI:** 10.3389/fncir.2012.00066

**Published:** 2012-09-24

**Authors:** Theofanis Karayannis, Natalia V. De Marco García, Gordon J. Fishell

**Affiliations:** Smilow Neuroscience, NYU Langone Medical Center, Neuroscience InstituteNew York City, NY, USA

**Keywords:** cortex, GABAergic, development, synaptic, caudal ganglionic eminence, homeostasis

## Abstract

Functional neuronal homeostasis has been studied in a variety of model systems and contexts. Many studies have shown that there are a number of changes that can be activated within individual cells or networks in order to compensate for perturbations or changes in levels of activity. Dissociating the cell autonomous from the network-mediated events has been complicated due to the difficulty of sparsely targeting specific populations of neurons *in vivo*. Here, we make use of a recent *in vivo* approach we developed that allows for the sparse labeling and manipulation of activity within superficial caudal ganglionic eminence (CGE)-derived GABAergic interneurons. Expression of the inward rectifying potassium channel Kir2.1 cell-autonomously reduced neuronal activity and lead to specific developmental changes in their intrinsic electrophysiological properties and the synaptic input they received. In contrast to previous studies on homeostatic scaling of pyramidal cells, we did not detect any of the typically observed compensatory mechanisms in these interneurons. Rather, we instead saw a specific alteration of the kinetics of excitatory synaptic events within the reelin-expressing subpopulation of interneurons. These results provide the first *in vivo* observations for the capacity of interneurons to cell-autonomously regulate their excitability.

## Introduction

Plastic changes in the mammalian cortex are important for normal function, adaptation, and neuronal circuit development. The importance of these changes is highlighted in pathological states when a significant perturbation results in impairment in plastic compensation that leads to epilepsy and/or neurodevelopmental disorders (Ramocki and Zoghbi, 2008; Walsh et al., 2008). Whether the homeostatic changes that should compensate during and after such a stressor event are always network-mediated or cell autonomous is an issue of intense research, which, due to technical limitations, has proven difficult to address *in vivo*. Hence, only a few studies have attempted to address this phenomenon using technically harder *in vivo* cell-autonomous experiments (Komai et al., 2006; Goold and Nicoll, 2010).

Homeostatic changes can take place at any level of a neuron's function from its intrinsic excitability to its synaptic inputs and outputs. Studies addressing both synaptic (Burrone et al., 2002; Turrigiano, 2008) and non-synaptic neuronal homeostasis have provided clues as to the possible mechanisms mediating such events (Burrone et al., 2002; Grubb and Burrone, 2010; Kuba et al., 2010; Nataraj et al., 2010). Experimental evidence has implicated two modifications utilized for synaptic plasticity: synapse specific long-term changes such as LTP and neuron-wide synaptic scaling (Turrigiano, 2008). Synaptic scaling, defined as a neuron's ability to regulate the number and/or strength of all synaptic inputs to achieve a constant synaptic output, has been attributed to both population and cell autonomous activities (Pozo and Goda, 2010). These results come mainly from *in vitro* research that has focused on the development of homeostatic excitatory changes occurring onto excitatory cortical cells in dissociated neuronal cultures (Burrone et al., 2002) and on *in vivo* cortical-wide activity manipulations, such as monocular deprivation (Nataraj et al., 2010). Collectively, these studies have suggested that the trigger for homeostatic synaptic changes arises from a change in the discharge of action potentials (AP), which in turn leads to changes in somatic calcium influx through voltage-gated calcium channels. Changes in calcium levels are postulated to provide the determining feedback that regulates both excitatory synapse number and the subunit composition of AMPA receptors onto excitatory cells (Beique et al., 2011).

Despite the wealth of information on the homeostatic changes undergone by the excitatory system, the possibility that similar changes occur in the inhibitory system have been less explored. Data obtained from dissociated hippocampal neurons show that inhibitory synapses are regulated through circuit mechanisms rather than in a cell autonomous manner (Hartman et al., 2006; Rannals and Kapur, 2011). This was postulated to work through both post- and pre-synaptic mechanisms. The former through an increase in the number of post-synaptic GABA_A_ receptors, whereas the latter is mediated through the release of BDNF, a well-known retrograde signal, which leads to an up-regulation of inhibitory input by acting on the presynaptic side (Hartman et al., 2006; Peng et al., 2010; Rannals and Kapur, 2011). Hence, homeostasis of excitatory and inhibitory synaptic inputs onto pyramidal cells can be achieved through regulation of spiking activity and/or a retrograde signaling from efferent synaptic partners (Harris, 2008). However, analysis of how homeostatic plasticity of inputs is regulated *in vivo* onto cortical inhibitory interneurons themselves has not yet been addressed.

GABAergic interneurons account for about 20% of the neurons within the cortex. The majority of these derive from the medial ganglionic eminence (MGE) and express parvalbumin (PV+) or somatostatin (SOM+). PV+ cells have been demonstrated to be centrally involved in plasticity during critical periods of cortical development (Sugiyama et al., 2008). In contrast, the majority of inhibitory interneurons present within superficial layers of the cortex have not been examined for their capacity for plasticity. These cells are derived from the caudal ganglionic eminence (CGE) and express reelin+, vasointestinal peptide (VIP+), and/or calretinin (CR+) (Miyoshi et al., 2010). Interestingly, in superficial layers, plastic changes that persist indefinitely are noticeable in pyramidal neurons only after postnatal day (P) 7, many days after the onset of deeper layer plasticity (Desai et al., 2002; Goel and Lee, 2007; Benedetti et al., 2009). P7 also corresponds to when superficially destined interneurons have completed their migration and are undergoing the peak in the development of their axo-dendritic tree, a process central to the establishment of their synaptic connectivity (De Marco Garcia et al., 2011; Miyoshi and Fishell, 2011). Their connections come primarily from intracortical/columnar excitatory and inhibitory cells of layers II/III, V, as well as a small amount from layer IV (Caputi et al., 2009; Xu and Callaway, 2009). Their proper connectivity and function is essential for the brain, since a failure of these processes has been shown to lead to epilepsy (Cobos et al., 2005).

The flexibility of neurons to modulate their function in accordance with changing contexts has proven remarkable (Marder and Taylor, 2011). However, when this adaptability fails because of either congenital or environmental factors, ailments occur (Walsh et al., 2008). Teasing out the variability in the homeostatic responses of different neuronal subtypes during development should provide insights into the etiology of a variety of childhood neurological disorders. To date, it has not been possible to address this issue *in vivo* due to the lack of the required methodology for targeting different cortical interneuron populations (Pozo and Goda, 2010). By taking advantage of a recent approach we developed, we have been able to examine the consequences of reducing neuronal excitability sparsely in superficial interneuron subtypes during development (De Marco Garcia et al., 2011). This strategy allowed us to study the mechanisms GABAergic interneurons utilize for activity regulation *in vivo*.

## Results

We focused our studies on the subset of GABAergic interneurons derived from the CGE since this population of neurons undergoes extensive activity-dependent development (De Marco Garcia et al., 2011). Indeed, reducing neuronal excitability within this population, by expressing the inward rectifying potassium channel Kir2.1 causes Re+ and Cr+ subtypes to acquire abnormal laminar positioning. In addition, these subtypes fail to develop proper axons and, therefore, exhibit reduced synaptic output. However, the consequences of developmental activity perturbations to the intrinsic excitability and incoming synaptic homeostasis on these interneurons have not been explored.

### Developmental time course of output of CGE subtypes

**Figure 1:**
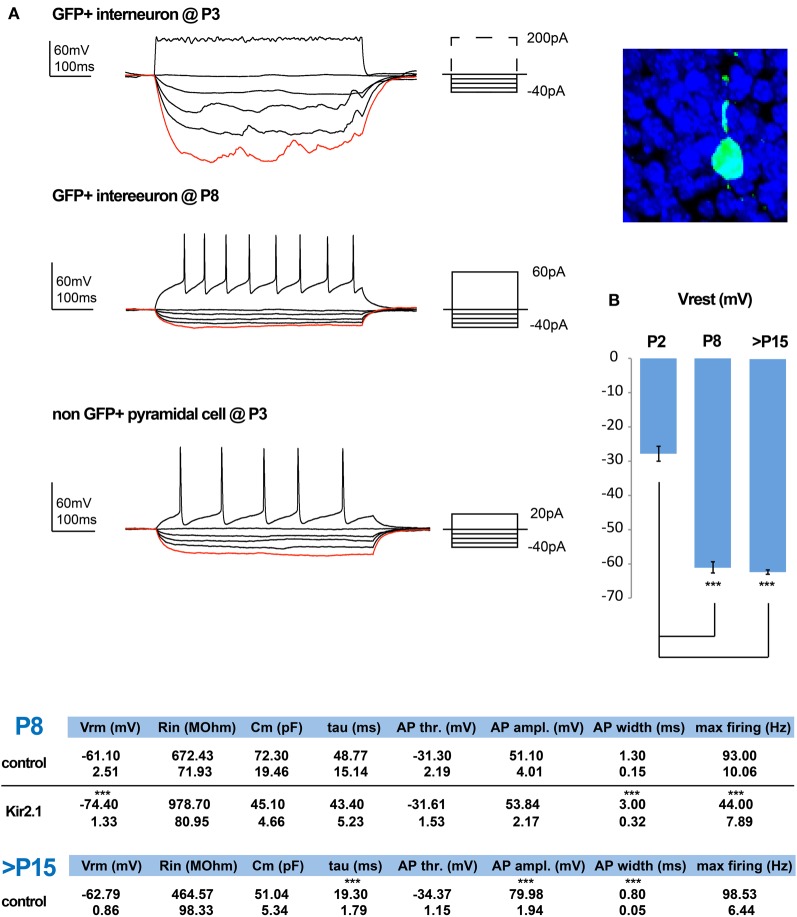
**Normal and activity-dependent developmental changes in the intrinsic electrophysiological properties of e15.5-born CGE-derived interneurons. (A)** Examples of recordings from e15.5-electroporated GFP+ interneurons within superficial layers at P3 (shown on the right) and P8. At P3 these neurons have a high input resistance (Rin) but lack the ability to generate action potential (AP) firing, even upon strong depolarization. In contrast, by P8 depolarization can induce APs and this is combined with a marked reduction in their Rin. In comparison, by P3 pyramidal cells are capable of generating proper APs. **(B)** The developmental profile of the resting membrane potential for GFP+ interneurons is shown. Tables showing the calculated values for the intrinsic properties of P8/9 in control and Kir.2.1 GFP+ interneurons, as well as for control cells that are >P15 (*n* = 6 and *n* = 9 and *n* = 21, respectively; asterisks denote significantly different to P2 for **(B)** and to control values for the tables using an unpaired *t*-test; ^***^ *p* ≤ 0.005).

To study the effect that the cell autonomous suppression of activity has on the integration of CGE-derived interneurons, we first examined the maturation of these interneurons under control conditions. We began by assessing the development of their intrinsic electrical properties, performing whole-cell patch-clamp recordings in cortical slices of *Dlx5/6-eGFP* electroporated mice (De Marco Garcia et al., 2011), at P2/3, P5, and P8/9. Labeled interneurons where recorded in current clamp and the resting membrane potential (Vrest) was determined with zero current injection as soon as electrical access into the cell was achieved. The cells became progressively more hyperpolarized with development, reaching their final Vrest values by P8/9 (Figure 1B, Tables). Subsequently, their potential for firing was assessed through the injection of depolarizing pulses of increasing intensity across these developmental ages. Before P8/9, interneurons failed to display fast overshooting sodium-dependent AP (Figure 1A) and instead some exhibited slower and shorter waveforms, reminiscent of calcium-mediated spikes (not shown). In contrast, superficial pyramidal cells born around e15.5 were capable of AP generation at least from P3 onwards. Nevertheless, by P8/9 all interneurons were capable of firing proper AP, albeit with lower amplitude and slower kinetics than the ones recorded at P15–21 (Figure 1, Tables). These results indicate that the occurrence of bona fide AP and hence synaptic output of e15.5 born CGE subtypes is initiated around P8/9, but further matures during the second postnatal week.

### Kir2.1-induced hypoexcitability markedly affects the maturation of intrinsic electrophysiological properties

We previously showed that expression of Kir2.1 initiated through E15.5 *in utero* electroporation leads to a drop in the Vrest of the targeted cortical interneuron population of approximately 13 mV when measured at P8 (De Marco Garcia et al., 2011). Here, we assessed the effect of reduced intrinsic excitability on the passive and active membrane properties of developing interneurons. Based on previous literature, we expected to find a homeostatic increase in the intrinsic excitability of these interneurons to compensate for the lowering of the Vrest. In contrast, we found no evidence that such compensation occurs. The input resistance (Rin) of Kir2.1-expressing interneurons, although on average surprisingly higher than in control cells, was not statistically altered (Kir2.1: 978.70 ± 80.95 MΩ, *n* = 9 vs. control: 672.43 ± 71.93 MΩ, *n* = 6). In addition, neither the capacitance nor the membrane time constant (τ) were found to be different between the two groups (Figure 1, Tables). Also, the active membrane properties, including the AP voltage threshold and amplitude were unaffected (control: −31.30 ± 2.19 mV and 51.10 ± 4.00 mV, *n* = 6 vs. Kir2.1: −31.61 ± 1.53 mV and 53.84 ± 2.17 mV, *n* = 9, respectively). In contrast, the AP waveform of Kir2.1-expressing interneurons was slower than in control interneurons with a half-width of 3 ± 0.32 ms vs. 1.3 ± 0.15 ms (*n* = 6 for controls and *n* = 9 for Kir2.1 cells; *p* = 0.0004). This difference likely underlies the reduced maximum discharge frequency of APs observed in Kir2.1-expressing interneurons compared to controls (Kir2.1: 44 ± 7.89 Hz, *n* = 9 vs. controls: 93 ± 10.06 Hz, *n* = 6; *p* = 0.0004) upon a 500 ms-long square current pulse. Thus, developing CGE interneurons do not exhibit a homeostatic increase in the AP discharge as previously reported for pyramidal cells *in vitro* (Burrone et al., 2002).

### Analysis of the development and maturation of synaptic inputs onto CGE subtypes

**Figure 2:**
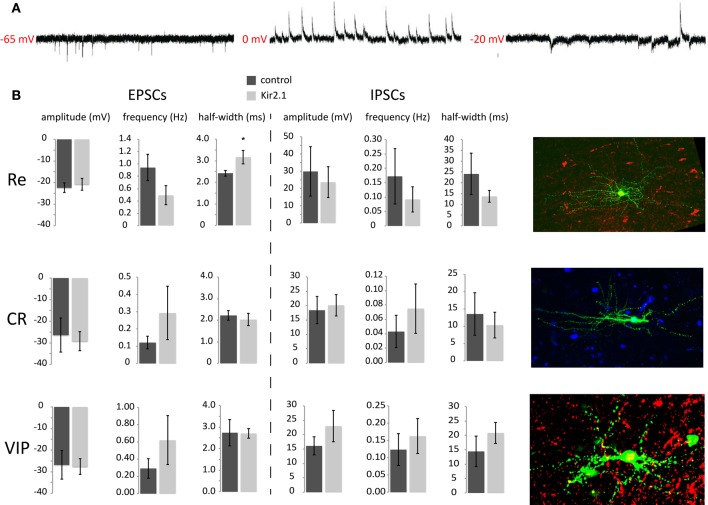
**Analysis of synaptic input onto e15.5-born CGE-derived GFP+ interneurons at P8/9 (A) Example traces of sEPSCs, sIPSCs and both, recorded at −65 mV, 0 mV, and −20 mV, respectively. (B)** Analysis of the sEPSC and sIPSC characteristics for the three different groups recorded, Re−, CR−, and VIP-positive interneurons without or with Kir2.1 expression. Representative images of the three different subtypes are shown on the right (pia is on the right). Green is the GFP, showing the axo-dendritic arborization of each one and red or blue is the respective marker for Reelin, Calretinin, or VIP (^*^p ≤ 0.05, unpaired *t*-test).

Previous studies showed that many of the inhibitory inputs that CGE-derived interneurons receive come from other upper layer CGE cells (Xu and Callaway, 2009). In this study we show that these cells are able to discharge overshooting AP from around P8 onwards, which in fact matches the time when they reach their final position in the cortex (Miyoshi and Fishell, 2011). Therefore, we started studying the maturation of synaptic inputs onto CGE developing interneurons at early (P8/9) and late (P17–21) timepoints by performing whole-cell patch-clamp recordings. The interneurons were held at different potentials in voltage-clamp to isolate and analyze both the spontaneous excitatory (sEPSCs) and inhibitory postsynaptic currents (sIPSCs) (Figure 2A) and were grouped based on their *post-hoc* immunolabeling for VIP, Cr, and Re and/or AP discharge characteristics (Figure 2B).

When analyzing the P8/9 recorded interneurons, we found that the frequency of sEPSCs in Re^+^ interneurons was higher than that of Cr^+^ but not statistically different to that of VIP^+^ interneurons (Re^+^ control: 0.94 ± 0.21 Hz, *n* = 8; Cr^+^ control: 0.12 ± 0.04 Hz, *n* = 5; VIP^+^ control: 0.29 ± 0.11 Hz, *n* = 4; *ANOVA with post-hoc Tukey's test p* < 0.05 Re^+^ vs. Cr^+^). Interestingly though, we do find a statistical difference in sEPSC frequency between Re^+^ and VIP^+^ interneurons at P15 onwards, with the former cells having more than the latter (Re^+^ control: 2.89 ± 0.59 Hz, *n* = 6; VIP^+^ control: 1.10 ± 0.24 Hz, *n* = 5; *p* = 0.03). In contrast, the amplitude (Re^+^ control: −22.47 ± 2.24 pA, *n* = 8; Cr^+^ control: −26.48 ± 7.85 pA, *n* = 5; VIP^+^ control: −27.68 ± 3.60 pA, *n* = 4), the risetime, decay time constant (tau), half-width, or charge of sEPSCs were not different among subtypes at P8/9 (*ANOVA with post-hoc Tukey's test p* > 0.05) (Figure 2B). The same is true for these measurements when comparing Re^+^ and VIP^+^ interneurons over P15 (Re^+^ control: −12.63 ± 1.41, *n* = 6; VIP^+^ control: −18.73 pA ± 3.00, *n* = 5). It is also interesting to note that the sEPSC characteristics change with age for the Re^+^ and VIP^+^ subtypes. Re^+^ cells show an increase in the frequency of sEPSCs with age but also a concomitant decrease of their amplitude (Re^+^ control frequency and amplitude at P8 vs. >P15; *p* = 0.01 *for both*). On the other hand, VIP^+^ cells show the same fold increase in the frequency of sEPSCs with age, but the amplitude does not reach statistical significance (VIP^+^ control frequency and amplitude P8 vs. >P15, *p* = 0.02 *for former and p* = 0.2 *for latter*). When sIPSCs were measured in the same cells at P8/9, no difference was found in the frequency or kinetics among the different groups (Figure 2B). Despite the sEPSC frequency differences, the EPSC/IPSC ratio was not altered.

One caveat of the above analysis is that it was restricted to the examination of spontaneous PSCs and therefore differences in PSC dynamics could be masked by multivesicular release events that would skew the distribution of values. To address this possibility at least in the case of inhibitory input, we recorded IPSCs as inward currents by changing the reversal potential for chloride in the presence and absence of tetrodotoxin (TTX) to unmask and compare mini (mIPSCs) to spontaneous IPSCs at P8/9. We found that although the IPSC frequency decreased upon TTX application, neither the amplitude nor the kinetics of the events were different (amplitude: −35.40 ± 10.95 pA vs. −39.29 ± 8.08 pA; risetime: 2.10 ± 0.40 ms vs. 2.10 ± 0.48 ms; halfwidth: 16.87 ± 2.37 ms vs. 18.20 ± 3.01 ms, sIPSC vs. mIPSC respectively, *n* = 4), indicating that CGE interneurons initially receive uniquantal IPSCs from presynaptic GABAergic cells. This mode of neurotransmission, as has been previously described for other synapses, may change to a multi-vesicular release at later stages (Riebe and Hanse, 2012). In the future it will also be of interest to examine the mode of excitatory synaptic release in this context.

### Kir2.1 expression causes selective defects to the development of synaptic inputs

Despite the lack of an upregulation of intrinsic excitability in maturing interneurons, homeostatic mechanisms may instead act by modulating the levels of afferent excitatory and inhibitory inputs. We reasoned that if these interneuron subtypes show a synaptic homeostatic response after Kir2.1 expression, it should be indicated by an upregulation of EPSC amplitude and/or frequency values and possibly a concomitant downregulation of those values for the sIPSCs as has been shown in different contexts by previous studies. However, when we recorded P8/9 interneurons, we found no significant differences in the amplitude (Re^+^ control: 30 ± 14 pA, *n* = 8 interneurons vs. Kir2.1: 24 ± 9 pA, *n* = 9; *p* > 0.05), frequency (Re^+^ control: 0.17 ± 0.1 Hz, *n* = 8 vs. Kir2.1: 0.09 ± 0.05 Hz, *n* = 9; *p* > 0.05) or kinetics of sIPSCs in Kir2.1-electroporated Re^+^ interneurons compared to controls (Figure 2B). Equally surprising, both the amplitude (Re^+^ control: −22 ± 2 pA, *n* = 8 vs. Kir2.1: −21 ± 3 pA, *n* = 9; *p* > 0.05) and frequency of sEPSCs were unaltered in Re^+^ Kir2.1-electroporated interneurons (Re^+^ control: 0.94 ± 0.2 Hz, *n* = 8 vs. Kir2.1: 0.49 ± 0.2 Hz, *n* = 9; *p* > 0.05) (Figure 2B). Nonetheless, we did observe that the kinetics of sEPSCs were significantly slower in Kir2.1 electroporated Re^+^ interneurons compared to controls (Figure 2B; EPSC monoexponential decay control: 2.7 ± 0.2 ms, *n* = 8 vs. Kir2.1: 4.0 ± 0.6 ms, *n* = 9; *p* = 0.05; half width of EPSCs control: 2.4 ± 0.1 ms, *n* = 8 vs. Kir2.1: 3.2 ± 0.3 ms, *n* = 9; *p* = 0.05; EPSC area of Re^+^ control: 83.0 ± 0.5 fC, *n* = 8 vs. Kir2.1: 103.0 ± 7.0 fC, *n* = 9; *p* = 0.04). These results suggest that neuronal activity may contribute to the proper maturation of excitatory synapses onto Re^+^ subtypes without having much effect on the overall excitatory innervation of these interneurons. In contrast, neither EPSCs nor IPSCs were affected with respect to amplitude, frequency, or kinetics in Kir2.1-expressing Cr^+^ and VIP^+^ interneurons compared to control cells. These findings are consistent with the normal development of dendritic trees in these two latter subtypes, as compared to the stunted dendritic growth observed in Re^+^ cells upon Kir2.1 expression (De Marco Garcia et al., 2011).

## Conclusion

This study examines the timecourse of CGE interneuron functional development, their integration into cortical circuits, and the role that early activity plays in these processes.

As we show herein, layer II/III pyramidal cells are born around E15.5 and by P3 have developed mature intrinsic electrophysiological properties and excitatory input (data not shown). By contrast, interneurons that undergo a protracted tangential migration from the caudo-ventral pallidum to the dorsal cortical plate (Marin et al., 2010) do not appear to produce AP or receive synaptic input until around P8. It therefore seems more likely that pyramidal cell activity would affect interneuron development through the excitation it provides rather than the reverse.

Here we examine the importance of excitability for the functional development of CGE-derived interneurons by expressing the inward rectifying potassium channel Kir2.1, which leads to a hyperpolarization of the cells and hence hypo-excitability. Pyramidal cells manipulated in this manner or upon sensory deprivation (Desai et al., 2002; Nataraj et al., 2010) show reduced Rin at P7 (Cancedda et al., 2007; Yoon et al., 2008) and a homeostatic increase in neuronal discharge after a few days in culture (Desai et al., 1999; Burrone et al., 2002). Unlike these findings on pyramidal neurons we observed an absence of typical activity-dependent compensatory mechanisms in all CGE subtypes. There was neither a rebound compensation in the intrinsic electrophysiological properties, nor a change in EPSC or IPSC amplitude and frequency, as would be expected based on previous studies of excitatory cells (Kilman et al., 2002; Turrigiano, 2008). Importantly though, most studies on pyramidal cells have been performed after their integration in neuronal networks and hence do not reflect developmental changes similar to the ones reported herein. It will be interesting to examine how the interneuron subtypes we studied respond to later alterations in activity.

In summary, the differences in the homeostatic changes previously observed in pyramidal cells and those we report here in interneurons might point to the differential ways by which these two groups develop, variations in how these distinct cell types compensate for alterations in activity, or a combination of the two. It might also be that some interneurons, such as the reelin-positive ones, first need to establish a set point with respect to their target values, before a wider variety of compensatory mechanisms to mediate homeostatic plasticity can occur (Davis, 2006). With the advent of ways to target and manipulate specific neurons such as select interneuron subtypes at discrete time points, there seems little doubt that considerably more insights into how neurons adjust their activity set points remains to be discovered.

## Materials and methods

### *In utero* electroporation

Pregnant Swiss Webster mice (Taconic) were electroporated at 15 days of gestation (E15.5) using a standard *in utero* electroporation technique. In brief, a timed pregnant mouse was anaesthetized and embryos were injected through the uterine wall in one lateral ventricle with 1–2 μl of DNA (3 μg μl^−1^). Fast green was used for visualization of the DNA solution. DNA was delivered by a glass needle operated with a mouth pipette. Five square 50-ms pulses at 40 V with a 950 ms interval were delivered with a 5-mm paddle electrode (CUY650P5, Protech International) using an electroporator (CUY21, Protech International). After electroporation, the uterus was placed back in the abdominal cavity and the mouse was sutured. The mice were kept on a warm plate (Fine Science Tools) through surgery to minimize hypothermia. After surgery, mice recovered in a humidified chamber at 30°C for 2–3 h. Mouse colony maintenance and handling was performed in compliance with the protocols approved by the Institutional Animal Care and Use Committee of the New York University School of Medicine.

The plasmids used in the electroporation experiments were generated using standard cloning techniques. The mouse *Kir2.1* and *eGFP* cDNAs were each individually cloned into a *Dlx5/6-Pmin-polyA* plasmid. Because eGFP expression was not detected in brains electroporated with a *Dlx-5/6-Kir2.1.ires.eGFP* polycistronic plasmid, the *Dlx5/6-eGFP* plasmid was co-electroporated with the *Dlx5/6-Kir2.1* plasmid at equivalent molar concentrations to ensure high levels of co-expression. The detection of similar levels of eGFP expression in *Dlx5/6-eGFP* and *Dlx5/6-eGFP/Dlx5/6-Kir2.1* electroporated interneurons indicates that transcription driven by this enhancer is not affected by Kir2.1 expression.

### Immunohistochemistry

Two hundred and fifty micro metre-thick vibratome sections were fixed for 2 h and incubated overnight at 4°C with selected antibodies. Sections were washed in PBS for several hours and incubated at 4°C overnight with donkey secondary antibodies (Jackson laboratories). Primary antibodies used in the experiments include rat anti-GFP (1:2000; Nacalai Tesque), mouse anti-Reelin (CR50) (1:500; MBL), rabbit anti-VIP (1:1000; Immunostar), mouse anti-calretinin (1:1500; Millipore Bioscience Research Reagents).

### Electrophysiology and analysis

Whole-cell patch-clamp electrophysiological recordings were performed on EGFP-positive and negative cells in acute brain slices prepared from P2–18 animals.

Briefly, animals were decapitated and the brain was dissected out and transferred to physiological Ringer's solution (ACSF) cooled down to 4°C of the following composition (mM): 125 NaCl, 2.5 KCl, 25 NaHCO_3_, 1.25 NaH_2_PO_4_, 1 MgCl_2_, 2 CaCl_2_, and 20 glucose. The brain was then glued to a stage and 250 μm slices were cut using a vibratome (Vibratome 3000 EP). The slices were allowed to recover in recording ACSF at room temperature for at least 45 minutes before recording. Acute slices were then placed in a recording chamber mounted on the stage of an upright microscope (Axioscope, Zeiss, Germany) equipped with immersion differential interference contrast objectives (5×, 40×) coupled to an infrared camera system (Zeiss), superfused at a rate of 1–2 ml/min with oxygenated recording ACSF and maintained at a temperature of 31°C. An EGFP filter was used to visualize the fluorescent interneurons in epifluorescence.

Whole-cell recordings were made from randomly selected EGFP-positive interneurons and non-labeled pyramidal cells located in upper layers (I–III) of the somatosensory cortex. Patch electrodes were made from borosilicate glass (Harvard Apparatus), had a resistance of 4–8 MΩ and for intrinsic electrophysiological properties and sEPSC recordings were filled with a solution containing (in mM): 128 K-gluconate, 4 NaCl, 0.3 Na-GTP, 5 Mg-ATP, 0.0001 CaCl_2_, 10 HEPES. For recording sIPSCs only the solution used was: 130 KCl, 10 K-gluconate, 10 HEPES, 10 Na_2_-phosphocreatine, 4 Mg-ATP, 0.3 Na-GTP, pH 7.3 with KOH. For acquisition of both sEPSCs an sIPSCs in the same cell the pipettes were filled with: 126 Cs-methylsulfonate, 4 CsCl, 0.3 Na-GTP, 4 Mg-ATP, 10 HEPES, 20 D-tris phosphocreatine. In all cases 5 mg/ml biocytin (Sigma) was added in the recording solutions.

Experiments were performed in current-clamp mode using the Axoclamp 2B (Molecular Devices) or the Axopatch 200B amplifier and in voltage clamp using the latter. Spontaneous synaptic currents were filtered at 3 kHz and recorded with a sampling rate of 10 kHz. Individually acquired sEPSCs and sIPSCs were recorded at Vh = −65 mV after application of kynurenic acid (3 mM) or a combination of CNQX (20 μM) and D-AP5 (20 μM) for the latter. When both were recorded from the same cell, the voltage was held at −65 mV for sEPSCs and at 0 mV for sIPSCs. The recorded files were analysed using Minianalysis software (Synaptosoft, Decatur, GA, USA). The synaptic values were obtained for the average trace after visual inspection of individual events. The decay time was calculated by fitting the average trace with a single exponential. Access resistance was always monitored to ensure the stability of recording conditions. Cells were only accepted for analysis if the initial series resistance was less than or equal to 40 MΩ and did not change by more than 20% throughout the recording period. The series resistance was compensated online by at least ~50% in voltage-clamp mode. No correction was made for the junction potential between the pipette and the ACSF.

Passive and active membrane properties were recorded in current clamp mode by applying a series of sub- and supra-threshold current steps and the analysis was done in Clampfit. The resting membrane potential (Vrest) was ascertained in current clamp right after rupturing the patch by applying zero current. All values presented in the manuscript are average ± standard error of the mean (SEM) and all the statistical values are obtained doing a standard Student's *t*-test, unless otherwise stated (^*^p ≤ 0.05, ^**^p ≤ 0.01, ^***^p ≤ 0.005).

All drugs were applied to the recording preparation through the bath. Salts used in the preparation of the intracellular recording solution and ACSF were obtained from Sigma-Aldrich.

### Conflict of interest statement

The authors declare that the research was conducted in the absence of any commercial or financial relationships that could be construed as a potential conflict of interest.
